# Alcohol, Anxiety, and Depressive Disorders

**Published:** 1996

**Authors:** Marc A. Schuckit

**Affiliations:** Marc A. Schuckit, M.D., is a professor of psychiatry at the University of California–San Diego, School of Medicine, and director of the Alcohol Research Center, Veterans Affairs Medical Center, San Diego, California

**Keywords:** AOD dependence, anxiety state, emotional and psychiatric depression, literature review, prevalence, children of alcoholics, treatment, prospective study, controlled study

## Abstract

Alcoholics frequently experience episodes of intense depression and/or severe anxiety. Depressed or anxious alcohol-dependent people often believe that they drink to relieve symptoms of sadness or nervousness. However, research does not unanimously support the prior existence of severe depressive or anxiety disorders as a usual cause of alcoholism. A review of recent literature (from family studies, prospective investigations, and studies of children of alcoholics) on the complex interaction between alcohol dependence and independent anxiety/depressive disorders reveals that if an association between alcoholism and anxiety/depressive disorders does exist, it likely operates in a relatively small subgroup of alcoholics at the same time. Psychological symptoms may carry a worse prognosis for alcohol-related problems, and these symptoms must be addressed early in alcoholism treatment.

The relationship between alcohol-use disorders and psychiatric symptoms is both clinically important and very complex (Brady and Lydiard 1993). As a typical depressant, alcohol affects the brain in many ways, and it is likely that high doses will cause feelings of sadness (i.e., depression) during intoxication that evolve into feelings of nervousness (i.e., anxiety) during the subsequent hangover and withdrawal. The greater the amounts of alcohol consumed and the more regular the intake, the more likely a person will be to develop temporary anxiety and depressive symptoms. As consumption increases even more, these symptoms also are likely to intensify.

It is, therefore, not surprising that more than one out of every three alcoholics has experienced episodes of intense depression and/or severe anxiety (Cox et al. 1990; Wilson 1988). These psychological conditions are often intense enough to interfere with life functioning, and the symptoms are often recognized by physicians and other health care providers as serious enough to require treatment. When depressed or anxious alcohol-dependent people are asked their opinions about cause and effect, they often reply that they believe they drink in order to cope with their symptoms of sadness or nervousness.

Two recent reviews, however, indicate that research does not unanimously support the prior existence of severe depressive or anxiety disorders as a usual cause of alcoholism (Allan 1995; Schuckit and Hesselbrock 1994). In this article, the term “depressive disorders” refers to an episode of major depressive disorder that significantly interferes with a person’s functioning over many weeks or months, and “anxiety disorders” refers to any of a number of serious and typically lifelong anxiety conditions (for further detail, see glossary, p. 86). Of course, when an alcohol-dependent person complains of severe depressive or anxiety symptoms (which might or might not indicate a long-term disorder), those conditions must be acknowledged and steps must be taken to help decrease them. If the psychiatric symptoms occur, however, as a consequence of the person’s consumption of high doses of alcohol (i.e., the complaints are alcohol induced), then the symptoms are likely to improve fairly quickly with abstinence. In this case, it is uncertain whether the longer term treatment of alcoholism requires additional aggressive therapies aimed at treating underlying depressive or anxiety disorders.

This article briefly reviews some of the recent literature on the complex interaction between alcohol dependence and the longer lasting anxiety or depressive disorders. The interactions between alcoholism and these disorders are evaluated by posing a series of questions, and the reader is encouraged to review the articles cited in the reference list. In keeping with the guidelines of *Alcohol Health & Research World*, review articles are emphasized. Readers interested in more detailed descriptions of the methods of particular studies, however, are referred to specific citations within those reviews.

## What are the immediate clinical implications of coexisting depressive and anxiety states among alcoholics?

As many as 80 percent of alcoholics report periods of sadness in their medical histories, with approximately one out of three alcohol-dependent men and women having experienced a severe depression that lasted for at least several weeks and interfered with his or her functioning (Brown and Schuckit 1988; Winokur 1983). Similarly, the majority of alcoholics admit to experiencing periods of nervousness, including at least 40 percent who have had one or more intense panic attacks characterized by a brief episode of palpitations and shortness of breath (Kushner et al. 1990).

An alcohol-dependent person who demonstrates such psychological symptoms needs more intense intervention and support than may otherwise be provided, and if not appropriately treated, the symptoms may carry a worse prognosis for alcohol-related problems. High levels of depression are especially worthy of concern, because the risk of death by suicide among alcoholics, estimated to be 10 percent or higher, may be most acute during these depressed states.

Once a person becomes deeply depressed, regardless of the cause, he or she may need to be hospitalized and provided with the appropriate precautions against suicide. These steps should be considered even if the patient’s depressive disorder is a relatively short-lived alcohol-induced state. Practitioners can counteract their patients’ depressive symptoms by providing education and counseling as well as by reassuring the patients of the high likelihood that they will recover from their depressions. Similarly, an alcoholic who experiences repeated panic attacks or other anxiety symptoms requires intervention for the anxiety, regardless of the cause. Alcoholics who experience high levels of anxiety or nervousness, including panic attacks, will likely benefit from education and reassurance as well as from behavioral therapies aimed at increasing levels of relaxation.

Most clinicians and researchers would agree that alcoholics experience high rates of anxiety and depressive symptoms and that these problems must be addressed early in treatment (Brady and Lydiard 1993). Increased debate, however, has focused on whether the depressive and anxiety disorders precipitated the patients’ alcoholism—in which case, longer term intensive treatments aimed at these psychiatric conditions might be required to ensure the optimum chance of recovery from alcoholism. Disagreement also exists about whether longer term independent treatment for depressive or anxiety diagnoses is required for the alcoholic person to achieve a normal level of life functioning. As previously mentioned, it is possible that many depressed or anxious alcoholics demonstrate mood or nervousness conditions *caused by* intoxication or withdrawal from alcohol; these psychiatric states are likely to improve markedly during the first several weeks to 1 month of abstinence. Thus, long-term psychiatric treatment does not appear to be required for alcohol-induced psychiatric conditions to be resolved (Brown and Schuckit 1988; Schuckit and Hesselbrock 1994).

## What evidence exists to support the conclusion that alcoholics have significantly higher-than-expected rates of long-term major depressive or anxiety disorders?

Certain theories give rise to the expectation that alcoholics might have high rates of long-term, independent anxiety and depressive disorders (Wilson 1988). For example, many psychological theories developed during the early-and mid-20th century proposed that people used high doses of alcohol to cope with the inappropriate resolution of more primitive phases of personality development, problems with sexuality or sex roles, and feelings of inadequacy or powerlessness (Vaillant 1995). Perhaps as a result of the influence of these theories, psychotherapists frequently reported deep-seated emotional difficulties or persisting psychiatric symptoms in alcoholics, even when alcohol-dependent people were sober.

As recently reviewed in the literature, some interesting data also support a possible relationship between longstanding anxiety or depressive disorders and alcoholism (Kushner et al. 1990; Kushner 1996). The findings include a higher-than-expected rate of anxiety or depressive symptoms among alcoholics or their relatives, and several studies indicate a possible increased rate of alcoholism among people presenting for treatment for depressive or anxiety diagnoses or among their relatives (Cox et al. 1990; Kushner 1996; Mason et al. 1996). The most consistent results relate to manic episodes, wherein manic-depressive patients show a small but significant increased risk for alcoholism (Winokur et al. 1993). Other data also suggest a greater-than-chance association between panic disorder (and perhaps social phobia) and alcoholism (Cowley 1992; Cox et al. 1990; Kushner 1996). These studies, however, do not clearly establish the intensity of the relationship between these psychiatric disorders and alcoholism (e.g., what percentage of alcoholics have independent anxiety disorders?), and the association of alcoholism to other mood or anxiety disorders is even less clear.

Although these studies raise important questions, researchers cannot draw definitive conclusions about the association between alcoholism and psychiatric disorders for a number of reasons. The major problem encountered in these studies involved the use of research methods that failed to address several important issues that might have explained the observed relationships (Allan 1995; Schuckit and Hesselbrock 1994). Specifically, some studies focused on drinking *patterns* rather than on alcohol dependence or described mood/anxiety *symptoms* rather than true psychiatric disorders. The distinction is important, because symptoms might be only temporary, whereas true psychiatric disorders are likely to require long-term and more intensive treatments, including psychotherapy and medication. Thus, few of the investigations offered assurance that an alcoholic or alcoholic’s relative actually had a long-term psychiatric syndrome rather than a temporary alcohol-induced condition.

In addition, researchers have considered whether alcoholism and some psychiatric disorders may have a genetic association—that is, whether they may be inherited together. Similar to alcoholism, most psychiatric disorders run in families and are genetically influenced. Thus, in reporting the rates of alcoholism or depressive/anxiety disorders among relatives of subjects, some studies may have overlooked the presence of both types of illnesses in the initial subjects or in the parents of the subjects’ relatives. The apparent association between alcoholism and these psychiatric syndromes may actually result from the marriages between individuals with the two separate disorders rather than a reflection of a single disorder in the family (i.e., the disorders would appear associated when in fact they occur independently in the same family). (For further information on the genetic association of alcoholism and psychiatric disorders, see the articles by Merikangas et al. and Woody, pp. 100–106 and pp. 76–80, respectively.)

Several separate lines of evidence cast doubt on the possibility that high proportions of alcoholics have severe, long-term depressive or anxiety disorders. These research approaches lead to three conclusions, discussed below.

### 1. Children of alcoholics (COA’s) do not have an increased risk for major depressive or anxiety disorders

Alcohol dependence has been shown to be genetically influenced and to run in families (Schuckit and Smith 1996). The disorder often develops when individuals are in either their twenties or thirties. Similarly, major anxiety disorders usually are apparent before age 30, and although major depressive disorders often have a later onset, they too are frequently observed before age 30. Therefore, if COA’s—people who carry a fourfold increased risk for the future development of alcoholism—often developed their alcohol dependence as a *consequence* of preexisting major psychiatric disorders, long-term studies of these young men and women should demonstrate a high rate of psychiatric syndromes before the onset of alcohol dependence.

Indeed, several disorders are more likely to be observed in COA’s than in control groups, including conduct problems, such as difficulties with discipline at home or in school (Schuckit and Hesselbrock 1994). As cited in our recent review, however, an evaluation by Hill and colleagues^1^ of 95 COA’s and control subjects at ages 8 to 18 showed no evidence of increased rates for depressive or anxiety disorders in the offspring of alcoholics (Schuckit and Hesselbrock 1994). That same review cited a second study of 283 COA’s and control subjects by Reich and colleagues^1^ that also reported no evidence for an increase in depressive disorders in COA’s, although evidence indicated a possible higher rate of anxiety symptoms. However, a prospective followup of 204 Danish COA’s and control subjects by Knop and colleagues^1^ demonstrated no differences between the 2 groups by age 20 with respect to either depressive or anxiety disorders. A subsequent followup of the Danish population revealed higher levels of anxiety disorders but not depressive episodes for the offspring of alcoholic parents, although by that age some of the symptomatology might already have resulted from high levels of alcohol or other drug (AOD) intake.

Schuckit and colleagues have studied the rates of psychiatric disorders in COA’s from a variety of perspectives. First, as cited in a review article, a survey of 18- to 25-year-old male students and staff at a university revealed no higher rates of depressive or anxiety disorders among COA’s compared with control subjects, a finding confirmed by a more intensive evaluation of men in a laboratory setting (Schuckit 1994). Second, the researchers conducted followup on 453 sons of alcoholics and control subjects who were tested in the laboratory at approximately age 20, thereby gathering data regarding the development of depressive, anxiety, and alcohol-use disorders during the subsequent decade (Schuckit and Smith 1996). In this followup study, although the sons of alcoholics were three times more likely to develop alcohol abuse or dependence, they showed no higher rates of major depressive disorders or major anxiety disorders during the followup period.

Only one notable study of COA’s has demonstrated a higher-than-expected risk for these major psychiatric disorders. As cited in a recent review, this investigation by Mathew and colleagues^1^ evaluated subjects at a relatively older age (i.e., at approximately age 40), and the research methodology did not adequately control for the possibility that the symptoms exhibited by these middle-age COA’s might have resulted from their higher alcoholism rates (Schuckit and Hesselbrock 1994). However, as pointed out by Kushner (1996), larger studies of COA’s who have passed the age of risk for most disorders will need to be conducted before final conclusions can be drawn.

### 2. Most prospective studies do not show higher rates of depressive or anxiety disorders in people who subsequently develop alcoholism

Vaillant (1995) has conducted a 40-year followup of 2 samples, one including more than 200 college men and the other including more than 450 blue-collar boys who were ages 11 to 16 at the time of the original study. Information was available on the subjects’ psychiatric symptoms and AOD-use patterns and problems, both at the time of enrollment into the study and at several points during the long-term follow-up. Despite finding that rates of alcohol abuse or dependence were relatively high in both samples, the researchers saw no evidence that preexisting depressive or anxiety disorders occurred at higher rates among those subjects who later developed alcoholism.

A recent review revealed similar results from other studies (Schuckit and Hesselbrock 1994). For example, a 10-year followup of young men and women who originally had been studied during their mid-teens by Ensminger and colleagues^1^ showed no close association between preexisting anxiety symptoms and AOD-use patterns in either sex. Similarly, in a study by Kammeier and colleagues,^1^ there was little evidence that preexisting psychiatric symptoms measured by a standard personality test predicted later alcoholism. From another perspective, as reported by Hagnell and Tunving,^1^ personal interviews conducted with more than 99 percent of the 950 males age 20 and older in a Swedish town revealed that alcoholics had a rate no higher than that of the general population for “neuroses,” a term likely to have encompassed both depressive and anxiety disorders. Also, an 18-year followup of 80 children who had experienced severe depressive episodes earlier in life revealed no evidence of an increased risk for alcoholism during the followup period (Harrington et al. 1990). Finally, Schuckit’s research group followed 239 alcoholic men 1 year after they received alcoholism treatment, and the data revealed no significantly increased rates of major depressive or anxiety disorders (Schuckit and Hesselbrock 1994). It is possible, however, that some of these studies might have excluded subjects with more severe anxiety or depressive disorders from the original samples, and consequently more work in this area is required (Kushner 1996).

### 3. When proper controls are used, some studies do not reveal high rates of long-term major depressive or anxiety disorders in relatives of alcoholics

A recent report from the Collaborative Study on the Genetics of Alcoholism (COGA) focused on 591 personally interviewed relatives of alcohol-dependent men and women (Schuckit et al. 1995). After controlling for potential alcohol-induced anxiety conditions in relatives, the lifetime risk for any major anxiety disorder in the male and female relatives of alcoholics was between 6.7 and 6.9 percent, rates not different from those expected in the general population. Neither male nor female relatives showed increased risks for obsessive-compulsive disorder, social phobia, panic disorder, and/or agoraphobia. A preliminary evaluation of the lifetime rates of major depressive disorders in 2,409 interviewed relatives of alcoholics revealed a rate of 17.5 percent, a figure that was almost identical to the rate observed in control families.

Similar results have been generated from some, but not all, studies of alcoholism in relatives of patients with severe anxiety disorders. For example, an evaluation of 1,047 adult relatives of 193 subjects with severe anxiety disorders revealed no increased risk of alcoholism among the relatives, with the exception of the relatives of those patients who had exceptionally early onsets of their psychiatric disorders (Goldstein et al. 1994). Nor did a review of several recent studies by Fyer and colleagues^1^ and Noyes and colleagues^1^ reveal high rates of alcoholism in relatives of people with social phobia or other anxiety disorders (Schuckit and Hesselbrock 1994).

Consistent with the generally negative results of these family type studies are the conclusions drawn from a recent study of 1,030 female-female twin pairs (Kendler et al. 1995). The researchers concluded that the genetic influences important in alcoholism appear to be relatively specific for that disorder and did not significantly alter the risk for additional psychiatric disorders, including major depression and major anxiety disorders. Another twin study by Mullin and colleagues^1^ showed no increased risk for *anxiety* disorders in identical twins of alcoholics with the exception of conditions (e.g., anxiety) that might have resulted from the alcoholism in the person’s twin.

It is important to remember, however, that certain studies show some overlap among depressive, anxiety, and alcoholic disorders in the same family. Many of these studies are mentioned in the Schuckit and Hesselbrock review, including the work by Merikangas and colleagues (1985). Other such studies are highlighted in the review by Brady and Lydiard (1993).

In summary, none of the three types of studies conducted (i.e., family studies, prospective investigations, and studies involving COA’s) proves an absence of a relationship between long-term anxiety or depressive disorders and alcoholism. As briefly discussed earlier in this article, the family studies are far from definitive because of difficulties in the methodologies used. It is also important to remember that some studies indicate a potential relationship between alcoholism and anxiety/ depressive disorders. In addition, alcoholism and these psychiatric disorders may operate together within some families, or individual instances may occur whereby a person develops alcoholism as a direct reflection of a preexisting psychiatric syndrome.

Conversely, the three types of studies highlighted in this section indicate that if an association between alcoholism and anxiety/depressive disorders does exist, it is likely to operate in a relatively small subgroup of alcoholics. More research is required before an adequate answer can be produced.

## What are the treatment implications of these findings?

The first conclusion to be drawn from this review is that many alcohol-dependent people are likely to present with depressive or anxiety symptoms that must be recognized and addressed. These problems contribute to an increased risk for suicide attempts, may be associated with more intense withdrawal symptoms, and may contribute to alcoholism relapse. Appropriate interventions for these psychiatric symptoms include forms of supportive psychotherapy, such as counseling or crisis intervention, and behavioral treatment, such as relaxation techniques and desensitization (Schuckit 1995).

Second, the possibility that a longer term anxiety or depressive disorder exists in an alcoholic must always be considered. Perhaps 10 percent of men and 10 to 20 percent of women in the general population develop severe anxiety or depressive disorders (Regier et al. 1990); therefore, it would be logical to expect that at least this proportion of alcoholics also would have similar syndromes. Identifying when an alcohol-dependent person has an independent or long-term major anxiety or depressive disorder requires gathering a careful patient history that searches for evidence of severe psychiatric symptoms either before the onset of severe alcohol-related problems or during a subsequent period of extended abstinence. Similarly, all alcoholics evidencing symptoms of severe depression or anxiety should be followed for approximately 1 month after abstinence to be certain that the depressive and anxiety symptoms are improving, because it is likely that severe symptoms remaining after abstinence for such a length of time may indicate a true independent depressive or anxiety disorder that requires longer term treatment.

However, treating most alcoholics’ depressive symptoms might not require the use of antidepressant medications. Although one recent small-scale study presented some promising results using these medications (Mason et al. 1996), most research performed in subjects without evidence of a long-term depressive disorder independent of their heavy drinking has found that antidepressant medications are unlikely to help the alcoholic maintain abstinence. These medications are not needed to help clear an alcohol-induced mood or depressive disorder. In fact, with abstinence the depressive symptoms are likely to improve in a shorter period of time than would be required for an anti-depressant to take effect (Brown and Schuckit 1988; Powell et al. 1995).

Similarly, in the absence of clear evidence of a long-term major anxiety disorder that predates the onset of alcoholism or that remains intense after an extended period of abstinence, few indications exist for using medications related to anxiety for alcoholics. Panic attacks that are likely to develop during alcohol withdrawal are also likely to diminish in frequency and intensity on their own without medications (Schuckit and Hesselbrock 1994). Because little evidence exists of an increased risk for obsessive-compulsive disorder among alcoholics, pharmacological treatments aimed at this severe anxiety condition also are inappropriate in the absence of additional evidence of an independent anxiety syndrome. Although more data are needed, at least one study indicates that buspirone, a medication useful for treating a general nervous condition called generalized anxiety disorder, may be helpful to some alcoholics, especially those with high levels of anxiety symptoms that persist after abstinence (Kranzler et al. 1994). However, not all studies agree on this point.

Fortunately, several important ongoing studies will help answer some remaining questions regarding the treatment of coexisting depressive or anxiety disorders in the context of alcoholism. The COGA investigation will gather more data regarding potential alcoholic subtypes and will continue to explore possible genetic linkages between alcohol dependence and major depressive and major anxiety disorders. Certain ongoing treatment studies also are further evaluating the potential usefulness of buspirone, some specific anti-depressants, and other medications that affect brain chemicals as potential components for treating alcoholism. Each of these studies is taking steps to evaluate the importance of these psychiatric medications while considering whether subjects’ depressive or anxiety syndromes are likely to be alcohol induced or may indicate longer term independent psychiatric disorders.

## Footnotes

1 For reviews of studies not cited in the reference list, see Schuckit and Hesselbrock 1994.
